# Anomalous Gallbladder: Atypical Acute Cholecystitis in a Patient With Phocomelia

**DOI:** 10.7759/cureus.116555

**Published:** 2026-09-19

**Authors:** Arif Akkok, Joanna Kondroski, Asli Akkok, Gagandeep Samra, Delcasse Joseph

**Affiliations:** 1 Orthopedic Surgery, Lake Erie College of Osteopathic Medicine, Elmira, USA; 2 General Surgery, Ross University School of Medicine, Miramar, USA; 3 Medicine, City University of New York (CUNY) School of Medicine, New York, USA; 4 General Surgery, St. John's Episcopal Hospital, Far Rockaway, USA

**Keywords:** anomalous gallbladder, atypical acute cholecystitis, biliary tract anomaly, congenital gallbladder anomaly, congenital limb deformity, phocomelia

## Abstract

Congenital anomalies of the gallbladder are exceedingly rare and, as a result, may complicate the diagnosis of biliary disease, especially when imaging findings do not align with clinical presentation. Phocomelia is a rare congenital malformation with multisystem involvement, though hepatobiliary anomalies are seldom reported. We present the case of a 45-year-old woman with phocomelia who presented with right upper quadrant pain and elevated bilirubin. Ultrasound and a hepatobiliary iminodiacetic acid scan were unremarkable, whereas computed tomography showed a fluid-filled structure along the superior hepatic surface, with the gallbladder absent from its expected fossa. Diagnostic laparoscopy demonstrated an ectopic suprahepatic gallbladder with a markedly elongated cystic duct and inflammatory changes. Laparoscopic cholecystectomy was performed with complete resolution of symptoms. This case highlights that anatomic variation, especially in phocomelia, may obscure typical imaging findings and warrants a high index of clinical suspicion when results are discordant.

## Introduction

Congenital anomalies of the gallbladder are rare but clinically significant, as they have the potential to complicate both diagnosis and surgical management of biliary disease [1,2]. Variations in gallbladder morphology, location, and cystic duct anatomy may obscure typical radiographic findings and create diagnostic uncertainty, especially in the acute setting. Missing these anomalies may delay treatment and lead to unexpected intraoperative challenges [1].

Ectopic gallbladder positioning, described in several locations, is an anomaly with important clinical implications [2-4]. Such positional variants may alter the radiographic presentation of common biliary conditions such as acute cholecystitis, causing discordance between clinical suspicion and imaging findings [3,4]. As a result, clinicians should remain alert to anatomic variants when standard diagnostic approaches are inconclusive.

Phocomelia is a rare congenital disorder in which the long bones of the limbs are severely shortened or absent, resulting in drastically shortened limbs. It may also be associated with other developmental anomalies [5]. Although it is not commonly associated with hepatobiliary variations, congenital anomalies such as phocomelia should prompt consideration of atypical anatomy in other organ systems.

## Case presentation

A 45-year-old woman with a past medical history of phocomelia presented to the emergency department with severe right upper quadrant and epigastric pain not associated with oral intake. She reported similar episodes in the past, along with current nausea and one episode of vomiting. At presentation, the patient denied fever, chills, chest pain, and shortness of breath. She was a former tobacco smoker who quit 20 years earlier.

On physical examination, the abdomen was soft with tenderness localized to the right upper quadrant. Murphy’s sign was positive. Limb defects included shortened bilateral upper extremities and a shortened right lower extremity, consistent with phocomelia. Laboratory evaluation demonstrated an elevated total bilirubin of 2.7 mg/dL (reference range, 0.2-1.2 mg/dL).

Abdominal ultrasound did not reveal cholelithiasis or biliary ductal dilation. Computed tomography (CT) of the abdomen and pelvis showed a fluid-filled structure with a thickened wall along the superior surface of the liver, with no gallbladder in the gallbladder fossa (Figures 1-2). Because of these discordant findings, a hepatobiliary iminodiacetic acid (HIDA) scan was performed. Imaging demonstrated normal hepatic uptake and excretion, with visualization of the gallbladder and normal biliary-to-bowel transit.

**Figure 1:**
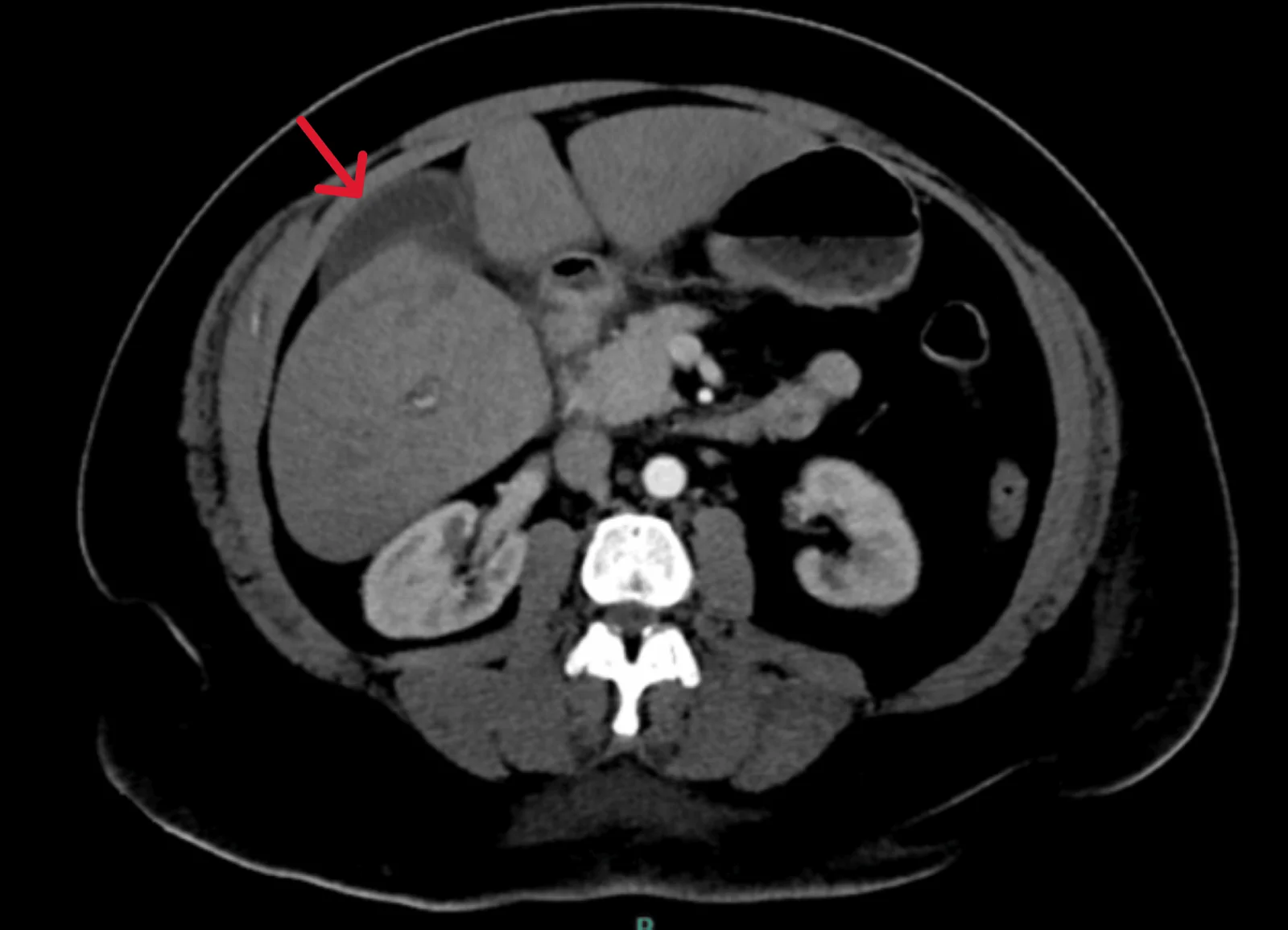
Axial CT image demonstrating a fluid-filled structure with a thickened wall along the superior surface of the liver with absence of the gallbladder in the gallbladder fossa. The red arrow indicates the ectopic gallbladder along the superior surface of the liver. CT: computed tomography

**Figure 2:**
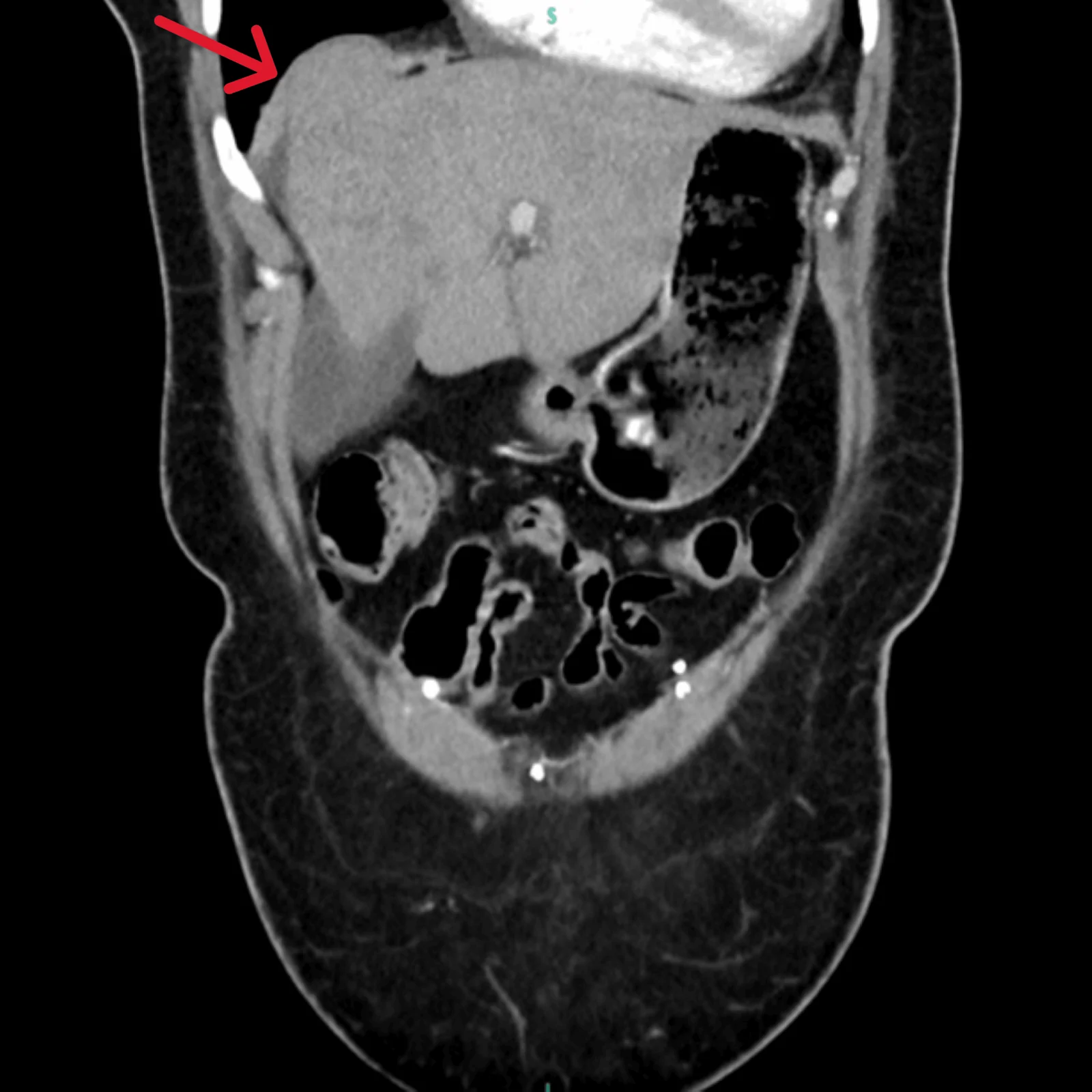
Coronal CT image demonstrating an ectopic, fluid-filled gallbladder with wall thickening along the superior surface of the liver and absence of the gallbladder within the expected gallbladder fossa. The red arrow highlights the ectopic gallbladder. CT: computed tomography

Despite inconclusive imaging and atypical findings for acute cholecystitis, the patient’s continuous symptoms and underlying phocomelia raised concern for an anatomic variant. The patient was taken for diagnostic laparoscopy and possible cholecystectomy.

Intraoperatively, the fluid-filled structure identified on CT was confirmed to be an ectopic gallbladder located along the superior hepatic surface (Figure 3). The gallbladder showed significant inflammation and edema. The cystic artery was not clearly identifiable. The cystic duct was markedly elongated, extending from the porta hepatis to the superior hepatic surface.

**Figure 3:**
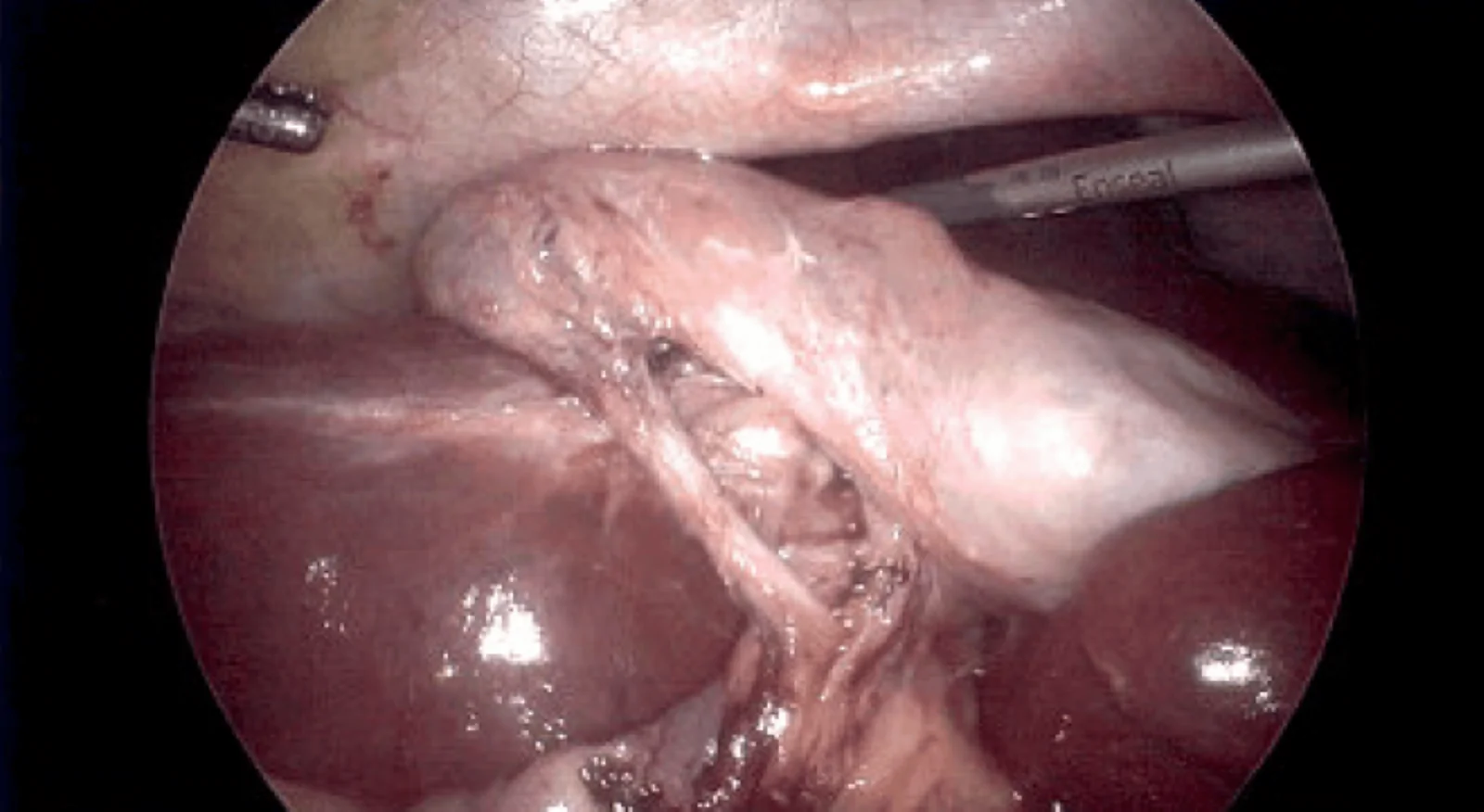
Intraoperative image demonstrating an ectopic gallbladder along the superior hepatic surface with an elongated cystic duct.

Following complete mobilization of the gallbladder from the hepatic dome, the cystic duct was clipped and transected, and laparoscopic cholecystectomy was performed without complication. The patient had an uneventful postoperative course with symptom resolution, and total bilirubin decreased to 0.8 mg/dL.

## Discussion

Phocomelia is a rare congenital malformation characterized by underdevelopment or absence of the long bones of the limbs, resulting in significantly shortened extremities. It is most notably associated with in utero exposure to thalidomide, a sedative drug historically prescribed to treat morning sickness in pregnant women, or as part of a genetic syndrome such as Roberts syndrome [5,6]. Phocomelia is generally associated with multisystem abnormalities, specifically involving the cardiovascular and genitourinary systems; however, hepatobiliary anomalies are rarely described in the literature [7].

In this case, ectopic positioning of the gallbladder along the superior hepatic surface, combined with a markedly elongated cystic duct, significantly altered the expected radiographic appearance of biliary pathology. This anatomic variant in the setting of phocomelia likely contributed to the discordant imaging findings, including a normal HIDA scan despite a clinical presentation suggestive of acute cholecystitis. Such variants can obscure typical imaging landmarks and reduce the diagnostic reliability of otherwise sensitive modalities [8-10].

Our case underscores that when imaging findings do not align with the clinical picture, particularly in patients with known congenital anomalies, reliance on imaging alone may delay appropriate diagnosis and management. In this patient, early consideration of diagnostic laparoscopy proved critical in establishing the diagnosis and facilitating definitive treatment.

Gallbladder anomalies associated with phocomelia are extraordinarily rare. The literature on hepatobiliary variations in this population is limited, with only isolated case reports of ectopic gallbladder positioning [8]. To our knowledge, this case represents one of the few reported cases of a suprahepatic gallbladder in a patient with phocomelia treated successfully with laparoscopic cholecystectomy, with the anomaly confirmed intraoperatively. This case emphasizes that minimally invasive surgical management remains feasible and effective, even when significant anatomic variation is encountered.

Surgeons should remain vigilant for atypical anatomy in patients with congenital disorders, specifically phocomelia, and be prepared to adapt their operative technique accordingly. Recognizing these variations is vital to avoid intraoperative complications and optimize patient care.

## Conclusions

This case highlights that the clinical presentation of acute cholecystitis may be obscured in patients with congenital disorders such as phocomelia. When clinical findings and imaging do not align, consider aberrant anatomy. The ectopic gallbladder and markedly elongated cystic duct observed in this patient underscore the importance of maintaining a high index of suspicion and adapting the surgical strategy to individual anatomy.

Individuals with phocomelia may exhibit a wider spectrum of anatomic variation than previously appreciated. Prompt recognition of these variants is essential, as reliance on imaging alone may delay diagnosis and management. Timely consideration of diagnostic laparoscopy in the proper clinical setting can facilitate definitive diagnosis and treatment.
